# Effect of wearing peripheral focus-out glasses on emmetropization in Chinese children aged 6–8 years: study protocol for a 2-year randomized controlled intervention trial

**DOI:** 10.1186/s13063-023-07799-8

**Published:** 2023-11-22

**Authors:** Li Shen, Wennan He, Weiming Yang, Weili Yan, Chenhao Yang

**Affiliations:** 1 Department of Ophthalmology, Children’s Hospital of Fudan University, National Children’s Medical Center, No. 399 Wanyuan Road, Shanghai, China; 2 Department of Clinical Epidemiology & Clinical Trial Unit, Children’s Hospital of Fudan University, National Children’s Medical Center, No. 399 Wanyuan Road, Shanghai, China

**Keywords:** Emmetropization, Myopia, Myopia defocus, Myopia prevention, Spectacle lens

## Abstract

**Background:**

Myopia is one of the most common eye diseases causing visual impairment and blindness, and the high prevalence in adolescents remains a major public health concern. Based on clinical studies using optical defocus to regulate ocular growth and refractive changes through visual feedback, we hypothesize that early wearing of peripheral myopic defocusing spectacles in children with high risk of myopia may slow the process of emmetropization and even prevent the onset of myopia by inducing more peripheral myopic defocus. The aim of this study is to investigate whether the wearing of peripheral focus-out glasses can be effective in delaying emmetropization in non-myopic children aged 6–8 years.

**Methods:**

The study is a 2-year randomized controlled trial. A total of 160 subjects will be randomized into the experimental group or the control group. The experimental group will be fitted with direct emmetropia with focus-out glasses (DEFOG) to guide the emmetropization process. The control group will not receive any treatment and will serve as a blank control group. The primary aim is to determine whether non-myopic children wearing DEFOG lenses are superior to those who do not receive any intervention on the progression of cycloplegic objective refraction over 2 years.

**Discussion:**

This is the first randomized controlled trial aiming at myopic prevention by non-invasive intervention in non-myopic children. This study aims to initially investigate whether wearing peripheral focus-out glasses can effectively delay the process of emmetropization in children aged 6–8 years with high risk of myopia, which might give potential clues for further exploration on early prevention of myopia.

**Trial registration:**

ClinicalTrials.gov NCT05689567. Registered on 10 January 2023.

**Supplementary Information:**

The online version contains supplementary material available at 10.1186/s13063-023-07799-8.

## Background

Myopia is a common eye disease that leads to visual impairment and blindness. Adolescents have a high rate of myopia, which is a significant public health issue. It is predicted that by 2050, 50% of the world’s population will be affected by myopia [1]. Unfortunately, the causes and development of myopia are not fully understood, and as a result, safe and effective prevention and treatment methods have not yet been developed.

During the developmental process of humans and animals, from being born with hyperopia to achieving emmetropia or myopia, there is a process called emmetropization [2]. If this process occurs too fast or too slow, it can lead to the occurrence of myopia or hyperopia [3]. Due to environmental factors and changes in living habits, the myopia rate of 6-year-old children in some parts of China has reached 15%. Moreover, the overall myopia rate of primary school students has exceeded 35%, indicating a trend of early onset, rapid development, and deep degree [4]. This means that most children complete the process of emmetropization too quickly, which inevitably leads to early myopia and contributes to a high prevalence of myopia.

Animal model studies showed that in the process of hyperopic focusing or after inducing experimental myopia, the use of a certain amount of myopic focusing can prevent the onset of experimental myopia or partially reverse myopia that has already occurred [5, 6].

Recent clinical studies indicated that for children who already have myopia, wearing multifocal soft lenses or peripheral myopic defocusing spectacles can significantly slow down the progression of their condition.

The BLINK study was an RCT study designed to investigate the effects of multifocal soft contact lens on the control of myopia progression in children. The three groups of myopic children were fitted with single-vision soft lens, multifocal soft lens with medium add power (+ 1.50 D), and multifocal soft lens with high add power (+ 2.50 D). Adjusted 3-year myopia progression was − 0.60 D for high add power, − 0.89 D for medium add power, and − 1.05 D for single-vision contact lens. The high add power group decreased the myopia progression by approximately 43% compared with the single-vision soft lens group [7].

In another RCT study about peripheral myopic defocusing spectacles, children were randomly assigned to wear defocus incorporated multiple segments (DIMS) or single-vision spectacle lenses (SV). Myopia progressed 52% more slowly for children in the DIMS group compared with those in the SV group. Likewise, children in the DIMS group had less axial elongation by 62% than those in the SV group. The result showed that simultaneous clear vision with constant myopic defocus can slow myopia progression [8].

Unfortunately, current myopia control measures are targeting children with myopia, by using low-concentration atropine [9], orthokeratology [10], peripheral myopic defocusing spectacles [8], and soft multifocal contact lenses [11], but lacking preventative measures for those without.

While the LAMP study and low-level red light study aim to provide early intervention for children who have not yet developed myopia, both treatments have certain limitations [9, 12]. Additionally, safety concerns related to their long-term use make it challenging to promote them on a wide scale in the near future [13].

To date, in order to reduce the rate of myopia and related complications, it is crucial to find effective methods early in children who do not have myopia. Therefore, delaying the onset of myopia in children may be the key to addressing the condition.

According to research, optical defocusing of eye growth and refractive changes can be regulated through visual signal feedback. Based on this, along with the clinical researches mentioned above and the safety of glasses, we believe that children can benefit from wearing peripheral myopic defocusing spectacles early on. This can help delay the process of emmetropalization and even prevent the onset of myopia.

To the best of our knowledge, a randomized trial aiming at the prevention of myopia by the non-invasive method is lacking. The prevalence of myopia is very high in Chinese school-age children, up to 70 ~ 80% [14], which supports the ethnic rationale and acceptability of using an intervention approach for preventing myopia in children population.

## Methods/design

### Aim of study

This study aims to investigate whether DEFOG lens can effectively delay the process of emmetropization in non-myopic children aged 6 − 8 years. The primary aim is to determine whether non-myopic children wearing DEFOG lenses have delayed progression of myopia drift of over 2 years compared with those who do not. Other changes such as axial length (AL), amplitude of accommodation (AMP), subfoveal choroidal thickness (ChT), and peripheral retinal refraction will also be compared during the study period.

### Study setting

The site of this study is the outpatient Department of Ophthalmology, Children’s Hospital of Fudan University. The address is No. 399 Wanyuan Road, Minhang District, Shanghai, China.

### Study design and recruitment

This is a randomized controlled superiority trial. A total of 160 children (aged 6 − 8 years) will be recruited and followed up for 2 years.

Potential participants will be recruited primarily via (1) offline advertising in outpatient clinics and (2) online advertising through domestic social media platforms.

The researchers will explain the purpose and content of the trial to the potential participants and their guardians, so as to be fully informed about the study content. Written consents of guardians will be obtained before enrollment. All the identifying information will be confidential and will be kept in locked cabinets that can only be accessible to the principal investigator and project director (Dr. Chenhao Yang and Dr. Weiming Yang). The protocol was approved by the institutional IRB on Nov. 2022 [approval no. (2022)315].

## Eligibility criteria

The following eligibility criteria for this trial are modified from those provided by the International Myopia Institute (IMI) [15, 16] and related studies [13, 14]: non-myopia children with high risk will be recruited.

1. Age of ≥ 6 and ≤ 8 years at enrollment
2. At least one parent’s spherical equivalent refraction ≤  − 3.00 diopters (D)
3. Spherical equivalent refraction (SER) under cycloplegia of + 0.50 to + 1.50 D
4. Astigmatism ≤ 1.00 D
5. Anisometropia ≤ 1.00 D
6. Best corrected visual acuity (BCVA) of 1.0 or better
7. Provision of consent written by the subject’s legal guardian
8. Willing and able to participate in all required activities of the study

Exclusion criteria: one will be excluded if any of the following criteria are met:

1. Any of the following abnormalities on the ocular surface: trachoma, pemphigoid, chemical injury, thermal burn, radiation damage, etc.
2. Eyelid abnormalities (such as entropion, ectropion, tumor, edema, blepharospasm, incomplete eyelid closure, severe trichiasis, and severe ptosis) that might affect eyelid function in either eye
3. Eye diseases such as strabismus, amblyopia, anisometropia, fundus diseases, and accommodation abnormality
4. Prior treatment of myopia prevention and control in either eye within 3 months before enrollment, including but not limited to atropine eye ointment, low-concentration atropine eye drops, orthokeratology, and low-level red-light therapy
5. Noncompliance with measurement at enrollment

## Intervention

1. Experimental group (DEFOG group): The experimental group will wear DEFOG lenses during the whole study period. Shanghai Guan Zhi Medical Technology Company will provide DEFOG glasses for this study. The bespoke company is commissioned by the researchers, who have no competing interest with the company, and the company will not be involved in the study itself during the whole process. The DEFOG lens is a custom-made plastic spectacle lens. It comprises a central plane optical zone and an asymmetrical peripheral myopic defocus zone with multiple segments of a positive power. This design simultaneously introduces myopic defocus on the peripheral retina and provides a clear vision for the wearers at all viewing distances. Baseline data including visual acuity, cycloplegic objective refraction, axial length, amplitude of accommodation, strabismus examination, pupil size, choroidal thickness, and peripheral retinal refraction will be measured at enrollment. A questionnaire survey will also be conducted to gather information in a clinical record form (CRF) about various behavioral and environmental factors that may affect the study, such as outdoor time, class time, time spent on extracurricular homework, usage of electronic devices, reading and writing habits, lighting conditions, and other relevant factors. The subjects are required to wear DEFOG lenses for at least 8 h a day, 5 days a week, and will be followed and examined at 6, 12, 18, and 24 months from baseline. In addition to ocular examinations, compliance and behavioral questionnaires will be recorded at any follow-up visit. During the follow-up period, if the cycloplegic SER is ≤  − 0.50D, the subject should stop wearing DEFOG lenses and start using other myopia prevention and control methods such as single focal glasses, low-concentration atropine eye drops, myopic focus-out glasses, orthokeratology, and multifocus soft glasses.
2. Control group: The control group will receive no intervention but general education about healthy reading and writing habits and will be a blank control, which reflects the real status of children today before the onset of myopia, with a follow-up schedule and measurements identical to the experimental group. Behavioral questionnaires will be assessed at each follow-up visit, including changes in reading and writing habits. If cycloplegic SER is ≤  − 0.50 D during the follow-up period, other myopia prevention and control methods will be initiated to control myopia progression.
3. Adherence: The adherence monitoring of DEFOG will be undertaken at every follow-up visit by interviewing the participants and guardians. The average time of wearing DEFOG will be recorded through a follow-up questionnaire by trained clinicians. Participants will be assessed whether they adhere to the intervention well or not according to the following rationale: (1) high compliance—the average wearing time is ≥ 5 days/week and 8 h/day; (2) moderate compliance—the average wearing time is 3–4 days/week or 6–7 h/day; (3) poor compliance—the average wearing time is < 3 days/week or < 6 h/day. For those with mild or poor compliance, investigators will communicate with the subjects and their guardians to consider any safety concern of wearing DEFOG, such as adverse events.

## Concomitant care and safety

Subjects will be allowed to subjectively withdraw from the trial at any time for no reason. Main adverse events like a decrease in visual clarity (defined as BCVA decreasing in 2 rows), dizziness, and discomfort will be recorded to evaluate the safety of wearing DEFOG at every follow-up visit, or subjectively reported by subjects or their guardians throughout the study period. Adverse events will be collected systematically in the same manner for each participant and recorded in the CRF. Unexpected harms will also be collected in the CRF. All the harms will be reported in trial publications, and the original CRF will also be uploaded to ClinicalTrials.gov if necessary. Investigators will address the signs and symptoms of the subjects in a timely manner and consider withdrawing subjects with severe adverse events from the study if necessary.

Throughout the entire study period, the use of myopia prevention and control medications or programs that may affect the analysis of outcome indicators, such as atropine eye ointment, low-concentration atropine eye drop, repeated low-level red-light therapy, and reversal flipper, is strictly prohibited. If participants used other myopia control measures or become myopic during the study period, assigned interventions will be discontinued but follow-up will be continued. No financial compensation will be involved in this trial.

## Study outcomes

### Rationale for outcome chosen

Myopia is an eye disorder characterized by light focusing in front of the retina due to excessive axial elongation of the eyeball. The evaluation of myopia is influenced by several factors, and in clinical settings, objective refraction (reflected by SER) under cycloplegia is considered more relevant for assessing changes in individuals with myopia. To avoid binocular interaction bias, only the right eye will be chosen for analysis of the study outcomes.

### Primary outcome

The primary outcome is the overall changes of cycloplegic objective refraction (reflected by SER) (right eye only, D) of the DEFOG group and the control group at 24 months from baseline. Objective refraction data will be examined using an auto ref/keratometer. SER is calculated by adding the sum of the sphere power with half of the cylinder power. Baseline data will be measured at enrollment. Other measurements obtained at follow-up visits are considered secondary outcome measures.

### Secondary outcomes

1. Changes of cycloplegic objective refraction (D): changes of cycloplegic objective refraction (reflected by SER) at different follow-up times (6, 12, and 18 months) from the baseline time (right eye only). Objective refraction data will be examined using an auto ref/keratometer
2. Cycloplegic objective refraction (D): cycloplegic objective refraction (reflected by SER) at 6, 12, 18, and 24 months (right eye only). Objective refraction data will be examined using an auto ref/keratometer
3. The occurrence of myopia: cycloplegic SER ≤  − 0.50 D at 6, 12, 18, and 24 months (right eye only). Objective refraction data will be examined using an auto ref/keratometer
4. Changes of axial length (AL) (mm): changes of AL at different follow-up times (6, 12, 18, and 24 months) from the baseline time (right eye only). AL will be examined using IOL Master
5. Axial length (AL) (mm): AL at 6, 12, 18 and 24 months (right eye only). AL will be examined using IOL Master
6. Amplitude of accommodation (AMP) (D): AMP at 6, 12, 18, and 24 months (right eye only). AMP will be examined using the lens test
7. Visual acuity: the BCVA at 6, 12, 18, and 24 months (right eye only)
8. Strabismus examination (△): strabismus examination at 6, 12, 18, and 24 months using a synoptophore
9. Changes of choroidal thickness (ChT) (µm): changes of ChT at different follow-up times (6, 12, 18, and 24 months) from the baseline time (right eye only). ChT will be examined by optical coherence tomography
10. Pupil size (mm): pupil size at 6, 12, 18, and 24 months (right eye only)
11. Peripheral retinal refraction (D): peripheral retinal refraction at 6, 12, 18, and 24 months (right eye only). Peripheral retinal refraction will be examined using an auto fundus camera

### Cycloplegia protocol

All of the refraction measurements will be obtained by a standard cycloplegia protocol. Ciliary muscle paralysis will be performed by using 1% cyclopentanone eye drops 2 doses in each eye, with an interval of 5 min between doses. The pupil size and light reflex will be examined after 30 min, and if the pupil is dilated to ≥ 6 mm and a light reflex is absent, cycloplegia will be deemed complete. Otherwise, the third drop of 1% cyclopentanone will be dropped in each eye. The optometry will be performed using an auto ref/keratometer.

### Participant timeline

**Fig. 1:**
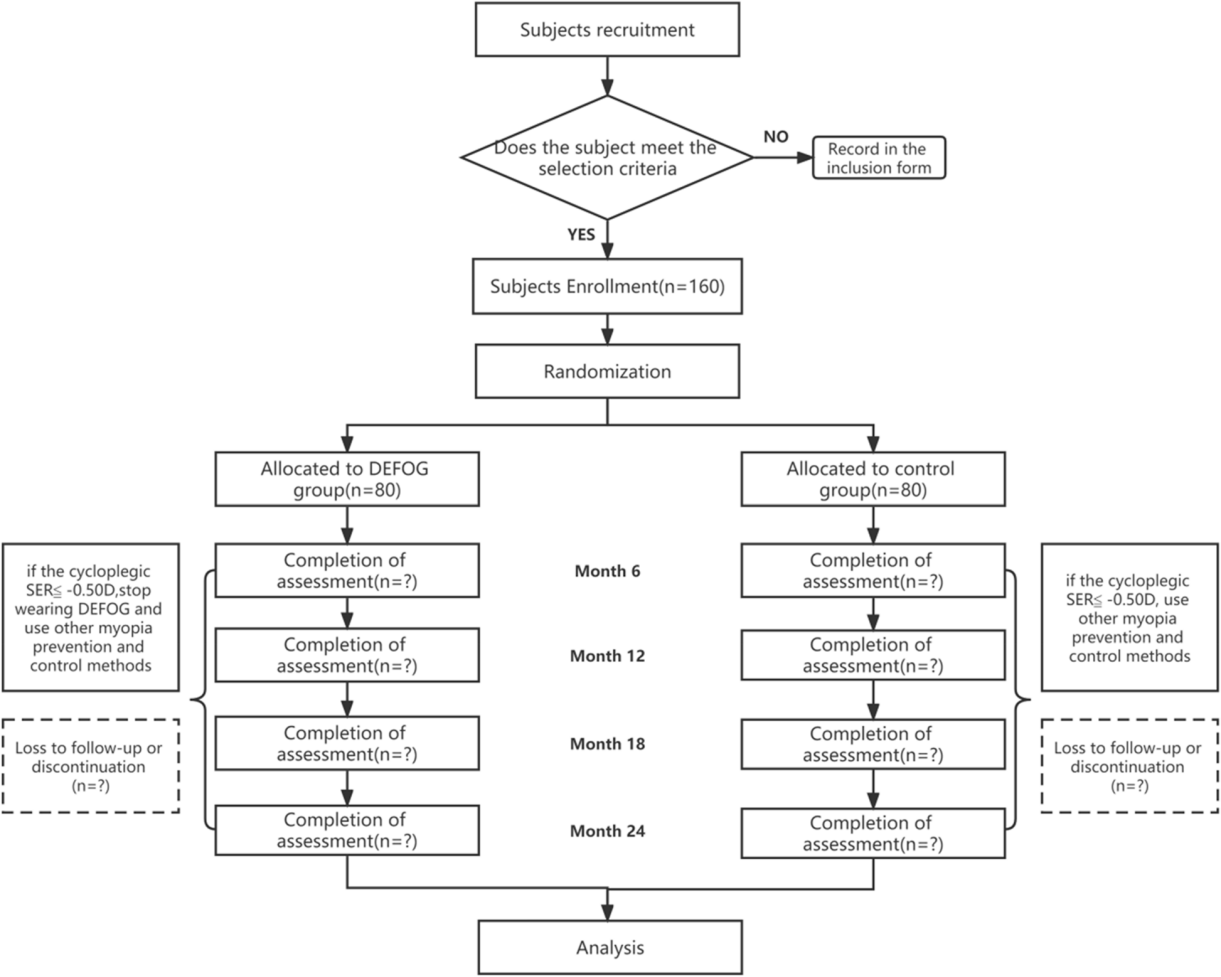
Flowchart of the DEFOG study

In this study, primary and secondary outcomes will be evaluated during the follow-up period according to the pre-determined schedule (Fig. 1 and Table 1).

**Table 1:** Schedule of activities

Procedure/measurements	Enrolment (− 2 to 0 weeks)	Allocation (0)	6 months (± 14 days)	12 months (± 21 days)	18 months (± 28 days)	24 months (± 35 days)
Informed consent	X					
Randomization	X					
Demographic data and medical history	X					
Allocation		X				
Compliance and behavior Habits			X	X	X	X
Visual acuity	X		X	X	X	X
Slit-lamp microscopic examination	X		X	X	X	X
Cycloplegic objective refraction	X		X	X	X	X
Axial length	X		X	X	X	X
Amplitude of accommodation	X		X	X	X	X
Strabismus examination	X		X	X	X	X
Pupil size	X		X	X	X	X
Fundus examination	X		X	X	X	X
Optical coherence tomography	X		X	X	X	X
Peripheral retinal refraction	X		X	X	X	X

## Sample size calculation

The primary outcome is the change in cycloplegic objective refraction (as measured by SER), as the rationale specified above. However, as this is the first preventative trial targeting non-myopic children by wearing lens, there is a lack of reference information on the preventative effect on refraction changes which is relevant to our study population. Considering changes in refraction are proportional to changes in axial length, the sample size calculation is alternatively referenced from a previous large trial in China, by using average change in axial length for non-myopic children aged 6–8 years over 24 months [17]. It is estimated that the axial length of the control group will increase by 0.6 mm ± 0.4 mm at the 24th month, and that of the experimental group will increase by 0.4 mm at the 24th month. Therefore, the value of Cohen’s *D* for sample size calculation is referred as the estimated effect between groups divided by baseline standard deviation in the control group, that is (0.6–0.4)/0.4 = 0.5, which will be subsequently converted into a suspected effect of change in refraction in this study. According to previous experience and the latest 2-year large trial in Shanghai, China [18], we conservatively assume an average change in refraction of − 1.00 D ± 1.00 D (i.e., 0.6-mm increase in axial length specified in the same study) over 2 years in children aged 6–8 years with a baseline refraction of 1.00 D ± 1.00 D in the control group. Thus, the estimated effect will be 0.5 × 1.00 + (− 1.00) =  − 0.50 D for change in refraction in the experimental group. This means that the sample size is fulfilled for us to observe at least − 0.50 D change between groups to reject the null hypothesis of no difference.

The sample size is calculated by using STATA15.1 (Stata Corp, TX, USA). Assuming a power of 0.80, a two-sided alpha of 0.05, and a 1:1 ratio between the experimental group and the control group, the estimated sample size for each group is 64 subjects. With an expected dropout rate of 20% based on previous clinical experience and other published trials [8, 12], the estimated sample size for each group is 80 subjects (i.e., 160 subjects in total).

## Randomization and masking

Block randomization with 1:1 ratio will be used to assign either DEFOG or blank control to enrolled subjects. The randomization sequence is generated by the independent statistic team at the Clinical Trials Unit of Children’s Hospital of Fudan University, using Stata 16.1 software (random seed number: 20230316). To ensure allocation concealment, each allocation sequence was placed in small, opaque, and sealed envelopes numbered and marked in order from 1 to 4 and were enclosed in a larger, opaque, and sealed envelope marked with the block number. The clinicians involved in this trial assess eligibility, enroll the subject after obtaining informed consent, and then contact the clinic coordinator who is independent of other research processes to obtain the allocation protocol for that subject. Block envelopes and the four enclosed small envelopes will be opened in order by the clinic coordinator.

The trial is unblinded due to the nature of the intervention. Subjects and clinicians responsible for enrollment and compliance assessment are aware of the group assignment, while the data analyst will not be informed of the assignment as the coding of group assignment will be hidden before then. To minimize the risk of observer bias due to unblinding, all ocular measurements will be performed by masked assessors (i.e., assessment-masked), who will not be aware of the allocation of the subjects and will not be involved in the intervention throughout the study. To avoid accidental unmasking, other assessment-masked strategies will also be included: (1) participants and guardians will be told not to reveal or talk about their group assignment with assessors during examination; (2) for the experimental group, DEFOG lens will be taken off in advance before they meet masked assessors and kept with unmasked clinicians until all examinations are done.

## Data collection and management

All the data of this trial, including the collection of outcomes, will be originally recorded in clinical record forms and then entered into the Microsoft Access database. Double data entry and checking will be completed on a wireless-enabled laptop by two trained operators who will not be involved in other procedures during the trial. The database will be coded and stored in a safe location that is only accessible to personnels involved in data collection. In the Access database, there are modules that allow for the identification of missing data, as well as quality assurance mechanisms. The data will be checked for data quality routinely and locked up when the last patient completes follow-up to achieve outcomes (i.e., 24 months follow-up).

This single-center trial will be primarily conducted by the Department of Ophthalmology in the Children’s Hospital of Fudan University, with statistical support from the Clinical Trial Unit (CTU) of the same hospital. An internal data monitoring committee (DMC) will be established and will consist of ophthalmologists from the Department of Ophthalmology who are not involved in the trial, statistical experts from the CTU team, and a senior statistician from a different institute for monitoring data completeness, safety information, adverse events, and so forth. No auditing will be performed through a professional organization.

Once the subjects are enrolled, retention efforts will be made to minimize loss to follow-up during the study, such as the following: (1) to not charge all examinations for all participants during the study period; (2) to develop a well-defined process in which all subjects will complete all examinations within 1 h, reducing the burden of follow-up visits; (3) to train and inform subjects and their families about the hazards associated with myopia, understanding how this trial may help their children, and increase the confidence of subjects and their families to complete the trial; and (4) to maintain a positive relationship with the subjects, using various means of communication such as WeChat, SMS, and telephone. Regular contact should be kept with a friendly attitude to establish a harmonious and trustworthy relationship. Additionally, it is necessary to strengthen the supervision and management of the subjects.

## Statistical analysis

Baseline characteristics of subjects will be presented as mean and standard deviation for continuous variables with normal or approximately normal distribution; otherwise, median values and interquartile range (IQR) will instead be calculated. Categorical variables (e.g., gender) will be described as proportions.

In the present study, the primary analysis will be performed following intention-to-treat analysis (ITT) strategy, which includes all randomized subjects regardless of protocol adherence. Repeated measures analysis will be used to determine changes from baseline over time and between the two study groups for both primary outcome and secondary continuous outcomes, by using the generalized linear mixed model (GLMM). The maximum likelihood method is used to estimate the mean difference between the two groups and their 95% CI, with Gaussian distribution and identity link function, group (group), time point (visit), and group × visit interaction as fixed effects and with subject ID as the random effect. Data from the right eye will be used for the analysis. To compare the prevalence rate of myopia between groups, a multivariable Poisson regression model will be applied to obtain the risk ratio and its 95% CI for myopia at the 24th month.

Sensitivity analyses will be conducted, including repeating the primary analysis based on per-protocol population, further adjusting age, sex, and baseline refraction which will be included as covariates in statistical models in the adjusted analysis. In addition, multiple imputation for handling missing data will be used if complete case analyses are not supported. Subgroup analysis will be performed for the primary outcomes and selected secondary outcomes, according to sex, baseline refraction grades, maternal and paternal myopic status, etc. Other strategies for additional analyses and missing data handling will be described in the statistical analysis plan and uploaded before the completion of this trial on ClinicalTrials.gov (NCT05689567). Interim analysis will not be considered in this trial design.

Statistical analyses will be performed using R4.1.2 (Vienna, Austria) and Stata 16.1 software (Stata Corp, TX, USA), and all statistical tests will be two-sided, with an alpha level of 0.05, and *P* < 0.05 will be considered a statistically significant difference.

## Ethics and dissemination

The trial protocol (V.2.0, 22 November 2022) is following the principles of the Declaration of Helsinki and has been approved by the research ethics board of the Children's Hospital of Fudan University [No.(2022)315]. It has been registered on 10 January 2023 at ClinicalTrials.gov (NCT05689567). Any modifications to the protocol which may have an impact on the conduct of the study and potential benefit of the patient or may affect patient safety, including changes of study objectives, study design, patient population, sample sizes, study procedures, or significant administrative aspects will require a formal amendment to the protocol. Such amendment will be approved by the research ethics board of the Children's Hospital of Fudan University prior to implementation and notified to the health authorities in accordance with local regulations. Informed consent will be obtained from potential participants by a senior doctor in the trial team, who has GCP certification. All project team members will be given access to the cleaned datasets, which will be password protected. The project principal investigator (Dr. Chenhao Yang) will have direct access to the original dataset. To ensure confidentiality, data dispersed to project team members will be blinded to any identifying participant information. Trial results will be published in peer-reviewed journals and will be disseminated to the media and the general public. No additional participant data or biological specimens will be collected that require further consent.

## Reporting of research protocol

This protocol is reported according to the SPIRIT 2013 reporting guidelines [19]. The checklist is filled out in the supplementary information of Additional file 1.

All items in accordance with the World Health Organization Trial Registration Dataset have been covered in ClinicalTrials.gov (NCT05689567, https://clinicaltrials.gov/ct2/manage-recs/register, registered on 10 January 2023), with a summary of the content shown in the supplementary information of Additional file 2.

## Discussion

The myopia rate of kindergarten children in some parts of China has reached 12%, and the overall myopia rate of primary school students has exceeded 69%, showing an early onset, rapid growth, and a deep degree [4]. However, this huge crisis seems to be not kept effectively under control until the myopia has already occurred. Driven by previous evidence that any correcting lens design that imposes peripheral myopic defocus such as orthokeratology, myopic focus-out glasses, and soft multifocal contact lenses will be effective in slowing the rate of myopia progression and ocular axial growth, how to slow down the process of emmetropization in non-myopic children may become the key point. In this way, this research will become a pioneering work that figures out a potential solution that might help narrow the gap in terms of myopia prevention. Given the high prevalence and dramatic increase in the myopia rate of Chinese children, it might benefit a large number of people if the efficacy of DEFOG has been proved.

The following are further considerations on the trial design:

1. The criteria of participants: Recent population studies have indicated that myopia is less prevalent in children under 6 years old (< 5%), but increases significantly after the age of 8 (30%) and can be as high as 50% by 10 years old [20–22]. Furthermore, there is a prospective study showing that there is no significant change in SER among kindergarten children aged 3–5 years old through a 1-year period, and there is no significant difference in SER between the 3-, 4-, and 5-year-old groups [23]. In a recent cross-sectional study, compared with 2020 (after COVID-19 home isolation), the prevalence of myopia among children aged 6 to 8 years in the 2021 screenings decreased, and the mean SER returned to pre-pandemic level. The refractive development in children aged 6 to 8 years may be most susceptible to environmental changes [24]. These remind us that 6–8 years old may be the key age range for our intervention in delaying emmetropization.
2. In this trial, we recruit non-myopic children who are at a high risk of having myopia within a short term, with parental myopia of SER ≤  − 3.00 D as inclusion criteria, which differs from the previous cluster RCT of non-myopic children [25, 26]. Parental myopia has a significant positive association with a child’s risk of developing myopia, as summarized in a recent meta-analysis [27]. Thus, these children will be more likely to benefit from the DEFOG intervention. High adherence to the trial will be more likely achieved due to the high willingness to prevent myopia from their guardians.
3. The two-year follow-up: Longitudinal analyses showed that became-myopic eyes were clearly different from emmetropic eyes 2 to 4 years before the onset of myopia in terms of refractive error, axial length, and relative peripheral refractive error. Eyes of became-myopic children differed in their rates of change between years, and the fastest interval for change in all three components was during the year before the onset of myopia. Became-myopic children had more myopia compared with emmetropes through 5 years after onset [28]. This suggests that there may be an opportunity for timely intervention in this short window.

This is the first randomized controlled study in delaying the process of emmetropization in non-myopic but at-high-risk children. Furthermore, this trial might not only provide reliable evidence to reflect the efficacy of the DEFOG design, but also give potential clues for further exploration on early prevention of myopia, by comparing the myopia onset between groups over 2 years.

### Supplementary Information

**Additional file 1.** Standard Protocol Items: Recommendations for Interventional Trials (SPIRIT) 2013 checklist: recommended items to address in a clinical trial protocol and related documents.

**Additional file 2.** Items from the World Health Organization Trial Registration Dataset.

## Data Availability

The datasets that will be generated and/or analyzed during the current trial will not be publicly available. Data sharing may be available for researchers from the corresponding author for research purpose only. The full research protocol and statistical analysis plan with statistical code will be first uploaded to ClincalTrials.gov or as supplementary files during journal publication if necessary.

## Abbreviations

DEFOG: Direct emmetropia with focus-out glasses
AL: Axial length
AMP: Amplitude of accommodation
ChT: Subfoveal choroidal thickness
IMI: International Myopia Institute
SER: Spherical equivalent refraction
BCVA: Best corrected visual acuity
ITT: Intention-to-treat analysis
PP: Per-protocol analysis
GLM: Generalized linear model
GLMM: Generalized linear mixed model
