# Supplementary material for: Effect of wearing peripheral focus-out glasses on emmetropization in Chinese children aged 6–8 years: study protocol for a 2-year randomized controlled intervention trial
Source: Trials. 2023 Nov 22;24:746. doi: 10.1186/s13063-023-07799-8 (PMC10666424; doi:10.1186/s13063-023-07799-8)
Supplement: Supplementary file 2 — Additional file 2. Items from the World Health Organization Trial Registration Dataset. [file 13063_2023_7799_MOESM2_ESM.docx]

| **Items from the World Health Organization Trial Registration Dataset** | |
| --- | --- |
| Data category | Information |
| Primary registry and trial identifying number | ClinicalTrials.gov NCT05689567 |
| Date of registration in primary registry | 10 January 2023 |
| Secondary identifying numbers | DEFOG |
| Source(s) of monetary or material support | Children’s Hospital of Fudan University |
| Primary sponsor | Children’s Hospital of Fudan University |
| Secondary sponsor(s) | Children’s Hospital of Fudan University |
| Contact for public queries | Chenhao Yang, MD ychben@hotmail.com |
| Contact for scientific queries | Chenhao Yang, MD Children’s Hospital of Fudan University, Shanghai, China |
| Public title | Focus-out Glasses on Emmetropization in Chinese Children |
| Scientific title | Effect of Peripheral Focus-out Glasses on Emmetropization in Chinese Children Aged 6-8 Years: 2 Years Randomized Clinical Trial |
| Countries of recruitment | China |
| Health condition(s) or problem(s) studied | Myopia prevention |
| Intervention(s) | Experimental: wearing of peripheral focus-out glasses≧8 hours a day and ≧5 days a week |
|  | Control: Blank control, without intervention |
| Key inclusion and exclusion criteria | Key inclusion criteria:   - Age: ≥6 and ≤8 years at enrollment. - At least one parent' s Spherical equivalent refraction≤-3.00D. - Spherical equivalent refractions (SERs) under cycloplegia: +0.50 to +1.50 diopters (D) |
|  | Key exclusion criteria:   - Any of the following abnormalities on the ocular surface which affect eyelid function in either eye - Eye diseases - Prior treatment of myopia control in either eye |
| Study type | Interventional,  Open-label, parallel assignment, randomized, assessment-masked controlled trial  Primary purpose: prevention |
| Target sample size | 160 |
| Recruitment status | Recruiting |
| Primary outcome(s) | Overall Changes of Cycloplegic Objective Refraction (spherical equivalent refraction, SER) (D) from baseline till the 24th month |
| Key secondary outcomes | Changes of Axial Length(AL)(mm), incidence of myopia, Visual Acuity, Changes of Choroidal Thickness (ChT) etc. |
